# Evaluation of two collagen conduits and autograft in rabbit sciatic nerve regeneration with quantitative magnetic resonance DTI, electrophysiology, and histology

**DOI:** 10.1186/s41747-018-0049-2

**Published:** 2018-08-08

**Authors:** Tina Jeon, Emil S. Vutescu, Eliana B. Saltzman, Jordan C. Villa, Scott W. Wolfe, Steve K. Lee, Joseph H. Feinberg, Sarah L. Pownder, Jonathan P. Dyke, Darryl B. Sneag

**Affiliations:** 1 Department of Radiology and Imaging, Hospital for Special Surgery, 535 E 70th Street, New York, NY 10021 USA; 2 Department of Hand and Upper Extremity Service, Hospital for Special Surgery, 535 E 70th Street, New York, NY 10021 USA; 3 Department of Rehabilitation Medicine, Hospital for Special Surgery, 535 E 70th Street, New York, NY 10021 USA; 4 Citigroup Biomedical Imaging Center, Weill Cornell Medical College, 516 E 72nd Street, New York, NY 10021 USA

**Keywords:** Trauma (nervous system), Nerve regeneration, Peripheral nerves, Sciatic nerve, Diffusion tensor imaging (DTI)

## Abstract

**Background:**

We compared different surgical techniques for nerve regeneration in a rabbit sciatic nerve gap model using magnetic resonance diffusion tensor imaging (DTI), electrophysiology, limb function, and histology.

**Methods:**

A total of 24 male New Zealand white rabbits were randomized into three groups: autograft (*n* = 8), hollow conduit (*n* = 8), and collagen-filled conduit (*n* = 8). A 10-mm segment of the rabbit proximal sciatic nerve was cut, and autograft or collagen conduit was used to bridge the gap. DTI on a 3-T system was performed preoperatively and 13 weeks after surgery using the contralateral, nonoperated nerve as a control.

**Results:**

Overall, autograft performed better compared with both conduit groups. Differences in axonal diameter were significant (autograft > hollow conduit > collagen-filled conduit) at 13 weeks (autograft vs. hollow conduit, *p* = 0.001, and hollow conduit vs. collagen-filled conduit, *p* < 0.001). Significant group differences were found for axial diffusivity but not for any of the other DTI metrics (autograft > hollow conduit > collagen-filled conduit) (autograft vs. hollow conduit, *p* = 0.001 and hollow conduit vs. collagen-filled conduit, *p* = 0.021). As compared with hollow conduit (autograft > collagen-filled conduit > hollow conduit), collagen-filled conduit animals demonstrated a nonsignificant increased maximum tetanic force.

**Conclusions:**

Autograft-treated rabbits demonstrated improved sciatic nerve regeneration compared with collagen-filled and hollow conduits as assessed by histologic, functional, and DTI parameters at 13 weeks.

**Electronic supplementary material:**

The online version of this article (10.1186/s41747-018-0049-2) contains supplementary material, which is available to authorized users.

## Key points

- Diffusion tensor imaging may be a biomarker for peripheral nerve regeneration
- Autograft shows improved sciatic nerve regeneration compared to collagen-based conduits
- Axial diffusivity correlated with measures of axonal diameter

## Background

Traumatic peripheral nerve injury is a potentially devastating ailment resulting in severe sensory and/or motor deficits. Neurotmetic injury, characterized by complete disruption of the nerve and its supporting structures, usually requires surgical intervention. Peripheral nerve injuries that cannot be repaired by a direct end-to-end, tensionless technique are addressed using either nerve autograft, the current standard of care [1, 2], nerve allograft, or a nerve conduit to bridge the gap. However, the use of autograft involves inherent morbidity at the harvested donor site, including the potential for neuroma formation, as well as increased operative time and cost due to a second surgical dissection [3–5].

Several nerve conduit types are currently in use or under investigation, and two of them were evaluated in this study. A Food and Drug Administration-approved conduit, NeuraGen®, (Integra LifeSciences, Plainsboro, New Jersey, USA; www.integralife.com) comprises a hollow inner structure and porous outer layer, both composed of a semipermeable type I collagen engineered to a specific pore size to allow for nutrient diffusion and nerve growth factor retention [6]. The other conduit studied was Nerbridge™ (Toyobo Co., LTD, Osaka, Japan; www.toyobo-global.com), an investigational product which comprises a coated collagen exterior with a porous polyglycolic acid (PGA) inner structure filled with collagen fibers. The inner structure composition of Nerbridge™, in theory, provides a scaffold to guide longitudinal propagation of regenerating nerve fibers [7].

Measurement of muscle recruitment with electrodiagnostic testing is the current standard of reference for assessing motor recovery following peripheral nerve injury and subsequent surgical intervention. It is operator-dependent and mildly invasive, however, requiring small needle placement into muscles. Diffusion tensor imaging (DTI) is a noninvasive magnetic resonance imaging (MRI) technique that has recently shown potential in quantifying peripheral nerve degeneration and regeneration in vivo by measuring the Brownian motion of water molecules [8, 9]. DTI indices reveal structural and orientation information of peripheral nerves including fractional anisotropy (FA), reflecting the degree of diffusion anisotropy of water molecules along axons, and the apparent diffusion coefficient (ADC), or magnitude of diffusivity. While axial diffusivity (AD) is inferred to reflect axon density, radial diffusivity (RD) is inferred to reflect myelin integrity [10, 11]. Alterations in DTI-derived metrics are believed to reflect changes in peripheral nerve microstructure and overall ‘nerve health’ [12]. Recent studies have demonstrated good correlation between DTI and functional and histologic parameters in assessing nerve regeneration in rodent models [8, 13] and rabbit models [14–17].

The goal of this study was to compare the efficacy of nerve regeneration between autograft and two different types of nerve conduits, Nerbridge™ and NeuraGen®, using DTI, electrodiagnostic testing, limb function, and histopathologic measurements.

## Methods

### Subjects

All procedures were performed in accordance with the National Institutes of Health Guide for the Care and Use of Laboratory Animals, with the approval from our Institutional Animal Care and Use Committee. A total of 24 adult male New Zealand white rabbits (weight 3.0–3.5 kg) were randomized into three surgical groups of eight rabbits each: autograft, hollow conduit (NeuraGen®), and collagen-filled conduit (Nerbridge™).

### Surgical procedure

**Fig. 1:**
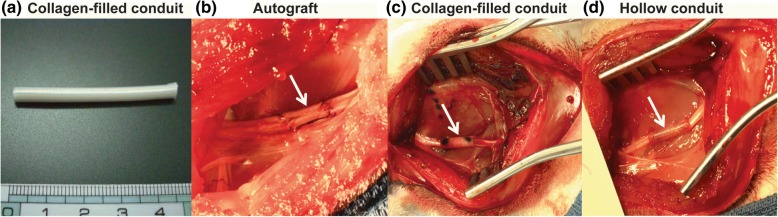
Location of the surgical site in the rabbit. The collagen-filled conduit (**a**) before implantation. Intraoperative photographs of autograft (**b**), collagen-filled conduit (**c**), and hollow conduit (**d**) surgically implanted to bridge the sciatic nerve gap at the midpoint between a line drawn between the greater trochanter and ischial tuberosity. The white arrows point to the location of the implanted conduits or autograft in vivo. Both conduits had a 3-mm inner diameter and were 12 mm in length

**Fig. 2:**
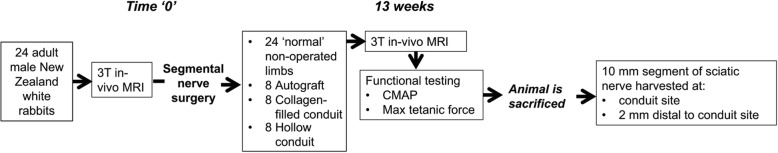
Timeline and study design. *CMAP* compound motor action potential, *MRI* magnetic resonance imaging

The nerve-gap surgical procedure was modeled after the description of Shin et al. [18]. Rabbits were induced intramuscularly with general anesthesia. The sciatic nerve was exposed along the far proximal, posterior right thigh, and a 10-mm nerve segment removed at the midpoint between a line drawn between the greater trochanter and ischial tuberosity. In the autograft group, the orientation of the 10-mm nerve segment was reversed and placed as an interposition graft with 9–0 nylon epineurial sutures (Ethicon Inc., Somerville, NJ, USA). For the conduit groups, the nerve gap was bridged with one of the two conduits (Fig. 1). MRI, histological, and functional testing analysis was performed blind to the type of surgical procedure performed. Figure 2 summarizes the timeline of the experimental methods.

### MRI acquisition and postprocessing

MRI of the bilateral thighs of the rabbits was performed in the prone position under the same anesthetic regimen as the surgical procedure, preoperatively (1–3 days before surgery) and postoperatively (13 weeks postsurgery), on a 3-T Siemens Magnetom Prisma unit (Siemens Healthcare, Erlangen, Germany) using an 8-channel flexible array coil positioned dorsally combined with 1–3 elements of a 32-channel spine array coil positioned ventrally under the thighs. A three-dimensional diffusion-weighted reversed fast imaging with steady state free precession sequence was acquired to localize the sciatic nerve, with the following technical parameters: echo time 2.72 ms; repetition time 9.45 ms; matrix 512 × 512; field of view 170 × 170 mm; slice thickness 0.9 mm, without gap; diffusion moment 90 s/mm^2^; slabs 96; bandwidth 465 Hz/pixel, acquisition time 8 min 5 s; number of excitations 1. A single-shot echo planar imaging DTI sequence was acquired with the following technical parameters: sensitivity encoding reduction factor 2; field of view 130 × 75 mm; matrix 76 × 44; slice thickness 1.7 mm, without gap; echo time/repetition time = 47/3400 ms; number of excitations 10; gradient directions 20, b-values 0 and 700 s/mm^2^; acquisition time 11 min 42 s.

**Fig. 3:**
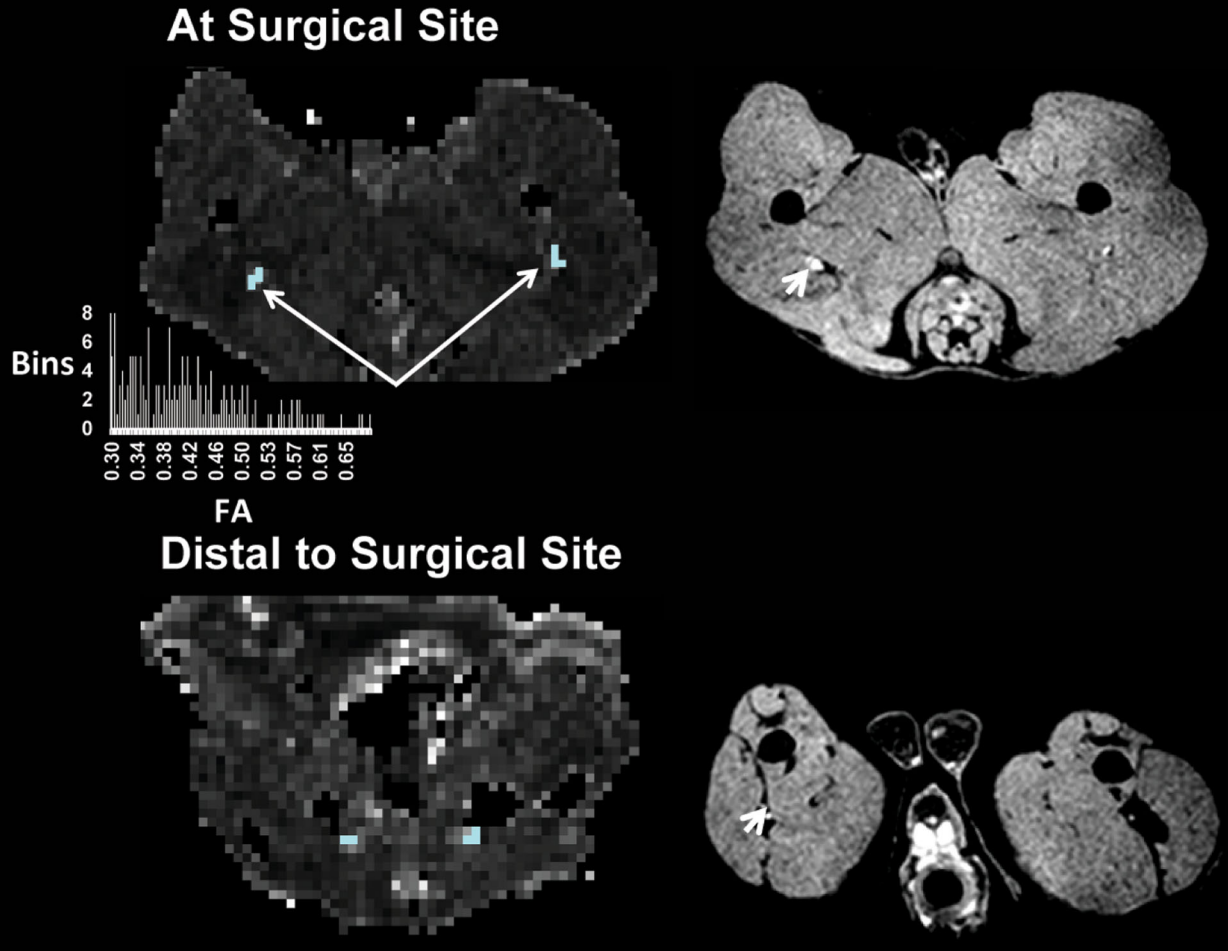
Segmentation of the sciatic nerve. Tensor fitting was performed with DTI Studio (www.mristudio.org) to obtain DTI maps after motion and distortion correction by linearly registering all gradient directions to the b = 0 map. Sciatic nerves were manually segmented using the fractional anisotropy (*FA*) map (left panel) as a mask by thresholding the nerve (minimum, maximum FA = 0.3, 0.8) and using the reversed fast imaging with steady state free precession sequence for anatomical nerve localization (right panel). A region-growing algorithm, using a custom code written in Interactive Data Language (www.harrisgeospatial.com), was employed to segment the entire nerve length. DTI measurements were extracted from voxels between the 25th and the 75th percentiles of the FA histogram to mitigate partial volume effects at the peripheral margins of the nerve. The surgical site was located proximal to the midpoint between a line drawn between the greater trochanter and ischial tuberosity (right panel, white arrows). Similarly, the distal site was located 3 mm distal to the surgical site on the FA map. Only the voxels inside the mask were included in the analysis

The DTI post-processing procedure is described in Fig. 3.

### Physiologic/functional testing

The rabbits underwent a nonsurvival motor testing procedure under anesthesia at the 13-week time point. Measurements were made with respect to percentage of the nonoperated nerve.

The animal weight was recorded immediately prior to surgery and prior to sacrifice. Anesthetized rabbits were examined for ankle contracture by measuring the angle between the anterior aspect of the tibia and dorsal aspect of the foot with the ankle in maximum passive plantar flexion.

To measure the compound motor action potential (CMAP), the sciatic nerve and its common peroneal and tibial divisions was exposed in the distal thigh and a miniature bipolar stimulating electrode needle was clamped on the exposed nerve. Recording electrodes were placed through the skin intramuscularly onto the surface of the tibialis anterior (TA) muscle and a ground surface electrode was placed on the foot for reference. Simulation duration was set at 0.02 ms and was performed at a stimulus intensity to obtain the maximum CMAP. CMAP latency and amplitude were measured using a Caldwell-Sierra EMG machine (Consolidated Neuro Supply Inc., Milford, OH, USA) with the Aurora Scientific software suite (Aurora, ON, USA; www.aurorascientific.com). The contralateral side was tested in an identical fashion.

The maximum isometric tetanic force (maximum tetanic) measurements were obtained to assess nerve potential force. The anesthetized rabbits were placed supine on a custom-built motor plate platform. A skin incision at the anterior aspect of the ankle was made to expose the TA muscle. The distal femur was stabilized to the platform by drilling into the distal femoral diaphysis (parallel to the plane of the tibial plafond) and stabilized with Kirschner wires (Pfizer Howmedica, Rutherford, NJ, USA). The distal TA tendon was secured to the level arm attached to the stimulator. The common peroneal nerve was then stimulated using a bipolar stimulator device (Harvard Apparatus, Holliston, MA, USA). For each muscle contraction, the electrical signal generated by the force transducer was processed using LabVIEW software (National Instruments, Austin, TX, USA). Maximum contractile force was calculated by subtracting the initial preload and repeated three times for each limb with a 5-min recovery interval.

At the conclusion of motor testing, all animals were sacrificed with 2.0 mL sodium pentobarbital 26%/isopropyl alcohol 10% administered through the lateral auricular vein.

The entire TA muscle from the operated and nonoperated limb was removed and weighed.

### Histopathological analysis

Following animal sacrifice, 5-mm segments of nerve at the surgical site (proximal to the midpoint between a line drawn between the greater trochanter and ischial tuberosity) and distal to the surgical site (3 mm distal from this location) were harvested from the operated limb and at the same anatomic location from the nonoperated limb. Nerve samples were fixed in glutaraldehyde for 24 h and washed in phosphate-buffered saline (pH 7.4).

Samples were then cut into 1-mm segments taken from the middle portion of each sample and postfixed in 1% osmium tetroxide for 6 h. The samples were dehydrated through ethanol solution and soaked in propylene oxide. They were then embedded individually in epoxy resin to polymerize overnight. Excess epoxy resins surrounding the sample were trimmed and cut into semi-thin transverse sections of 2 μm in thickness and stained with toluidine blue dye.

Transverse sections were viewed under a light microscope, and randomly sampled microscopic visual fields were captured using image analysis software (BIOQUANT Osteo II, BIOQUANT Image Analysis Corporation, Nashville, TN, USA) to quantify myelinated axonal density and diameter.

### Statistical analysis

Spearman correlation coefficients between DTI measurements and histology, DTI and motor testing, and between motor testing and histology were calculated along with sample size and Fisher-approximated 95% confidence interval, stratified by treatment group and site. A linear mixed-effects regression model was used to compare DTI metrics of the operated and nonoperated nerve as well as to measure between group differences for all three groups for histology, motor testing, and DTI. The Omnibus *F* test and pairwise group comparisons were calculated with Bonferroni correction for multiple comparisons. Omnibus group comparisons were conducted with the Kruskal-Wallis test and pairwise group comparisons were analyzed with the Steel-Dwass-Critchlow-Fligner test [19]. An alpha value of < 0.05 indicated significance, and all analyses were conducted in SAS 9.4 (SAS Institute Inc., Cary, NC, USA).

## Results

Following the animal sacrifice, gross inspection of the surgical sites revealed prominent segments of scar tissue encasing the sciatic nerve for all groups. All non-nerve tissue was carefully discarded prior to histological examination. Two hollow conduits were still intact at 13 weeks while all of the collagen-filled conduits dissolved completely.

### Histopathologic findings

**Fig. 4:**
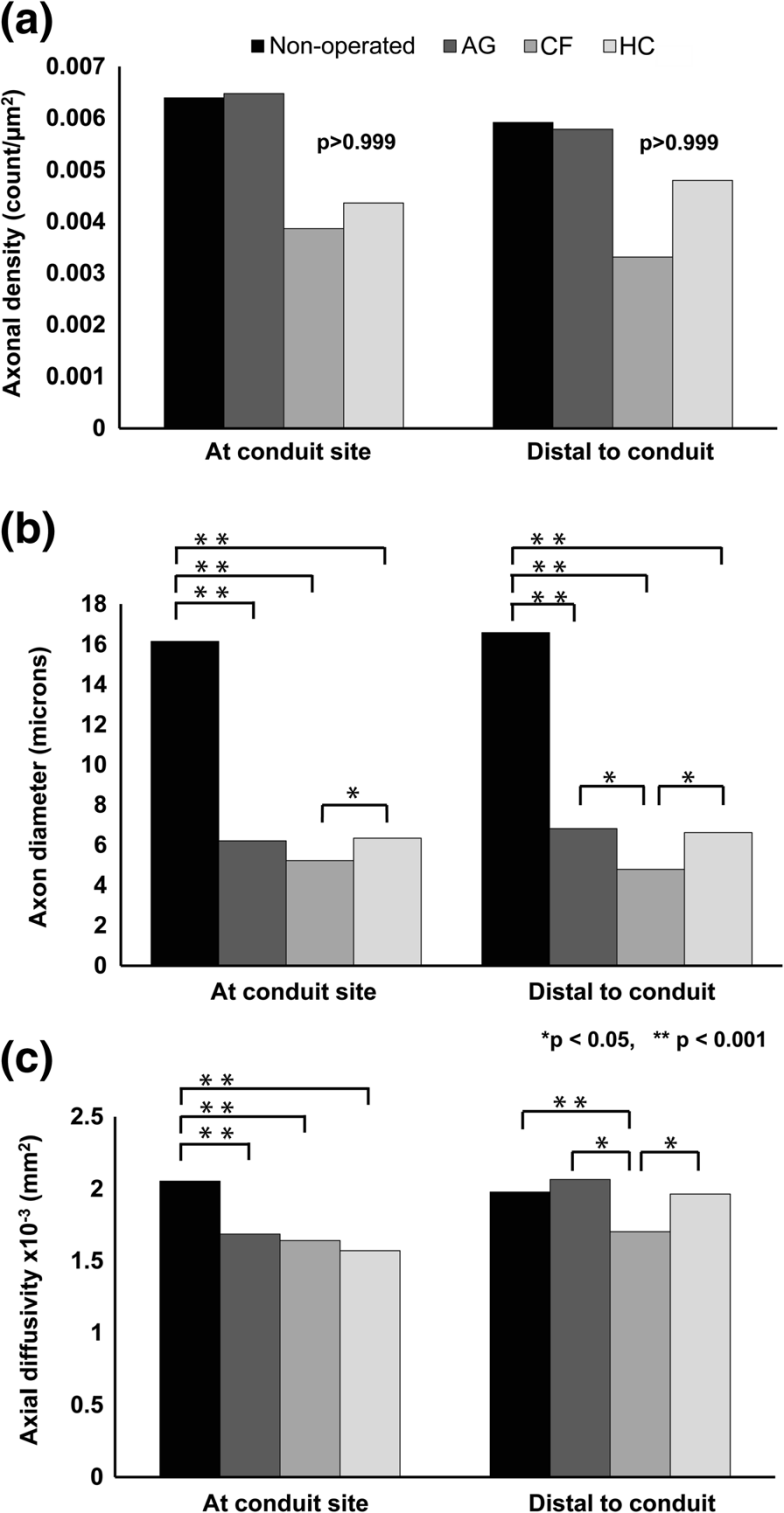
Axon density, diameter, and axial diffusivity group differences at and distal to the surgical site. Myelinated axon density (**a**), diameter (**b**), and axial diffusivity (**c**) of the three different nerve surgeries, autograft (AG), hollow conduit (HC), and collagen-filled conduit (CF), compared with the nonoperated nerve. There were no significant differences between groups for axonal density either at or distal to the surgical site. The asterisks indicate statistical significance as indicated

**Fig. 5:**
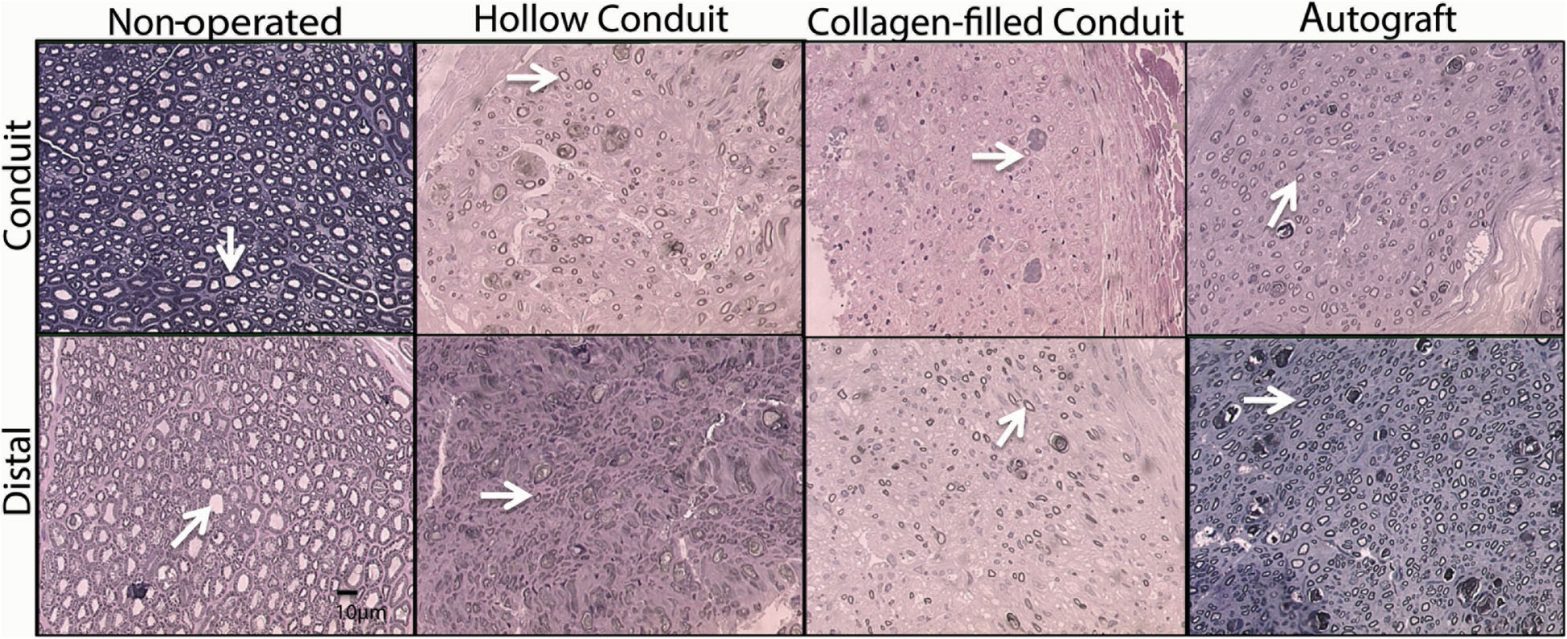
Micrographs of orthogonal sections of the sciatic nerve 13 weeks after segmental nerve surgery. Histologic sections of the sciatic nerve distal to and at the surgical site 13 weeks postsurgery of autograft, collagen filled conduit, and hollow conduit with toluidine staining at 100× magnification. For comparison, the sciatic nerve of the nonoperated limb is shown in the left panel. The white arrows indicate myelinated axons. Note the visually decreased diameter of the operated nerves compared with normal controls. Due to processing difficulties, seven samples were not included in the histology analysis: two autograft at the conduit site, one autograft distal to the graft site, two collagen-filled at the conduit site, and two hollow conduit distal to the conduit site. Color differences are due to staining/drying of toluidine blue

Group differences were not statistically significant for axonal density (Fig. 4a). Significant differences in axonal diameter at the surgical site were found between the hollow conduit and collagen-filled conduit (*p* = 0.014). Distal to the surgical site, significant differences in axonal diameter between autograft and hollow conduit (*p* = 0.001) and hollow conduit and collagen-filled conduit (*p* < 0.001) were found (Fig. 4b). Representative histologic sections of the sciatic nerve for all groups are shown in Fig. 5.

### Physiologic/functional testing

Differences in physiologic and functional measurements were not statistically significant between conduit groups (Table 1). Although not significant, the maximum tetanic force percentage increase (29.8%) for the collagen-filled conduit was almost equivalent to that of autograft (35.3%). However, there was high variance in both group measurements.

**Table 1:**
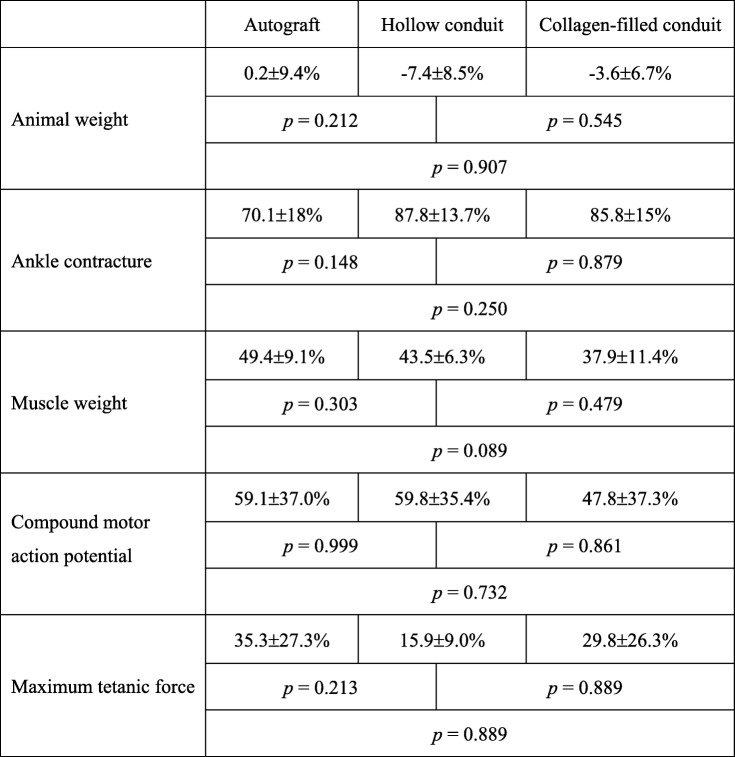
Physiological/functional testing measurements for all groups

Data are reported as mean ± standard deviation

Animal weight is reported as percentage weight loss/gain 13 weeks postoperatively

Ankle contracture, muscle weight, compound motor action potential, and maximum tetanic force are reported as percentage of the nonoperated nerve

All *p* values were obtained according to the Steel-Dwass-Critchlow-Fligner method

### DTI metrics

Values for DTI metrics at 13 weeks are summarized in Table 2. FA, ADC, and RD were not significantly different between groups both at and distal to the surgical site. AD was significantly greater in autograft compared with collagen-filled conduit (*p* = 0.001) and greater in hollow conduit compared with collagen-filled conduit (*p* = 0.021) distal to the surgical site (Fig. 4c). AD group differences positively correlated with axonal diameter group differences between autograft and hollow conduit (*p* = 0.001) and between hollow conduit and collagen-filled conduit (*p* < 0.001). Differences between DTI metrics of the nonoperated limb at the preoperative and postoperative time points were not significantly different for all rabbits irrespective of group (*p* = 0.751–0.909). DTI measurements of the entire length of the sciatic nerve revealed no significant group differences between all groups (Additional file 1: Figure S1).

**Table 2:**
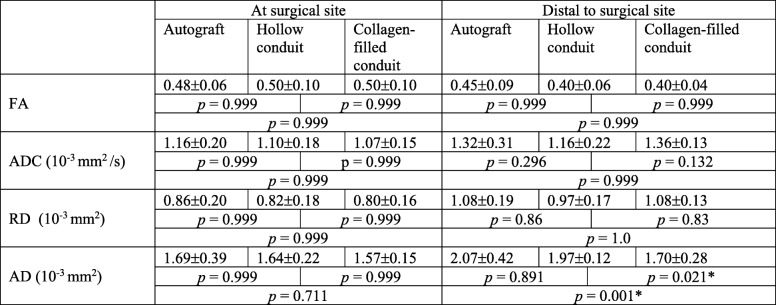
Diffusion tensor imaging metrics (FA, ADC, RD, and AD) for all groups at and distal to the surgical site

Data are reported as mean ± standard deviation

All *p* values were obtained with a pairwise *t* test

*AD* axial diffusivity, *ADC* apparent diffusion coefficient, *FA* fractional anisotropy, *RD* radial diffusivity

* Statistical significance after Bonferroni correction

**Fig. 6:**
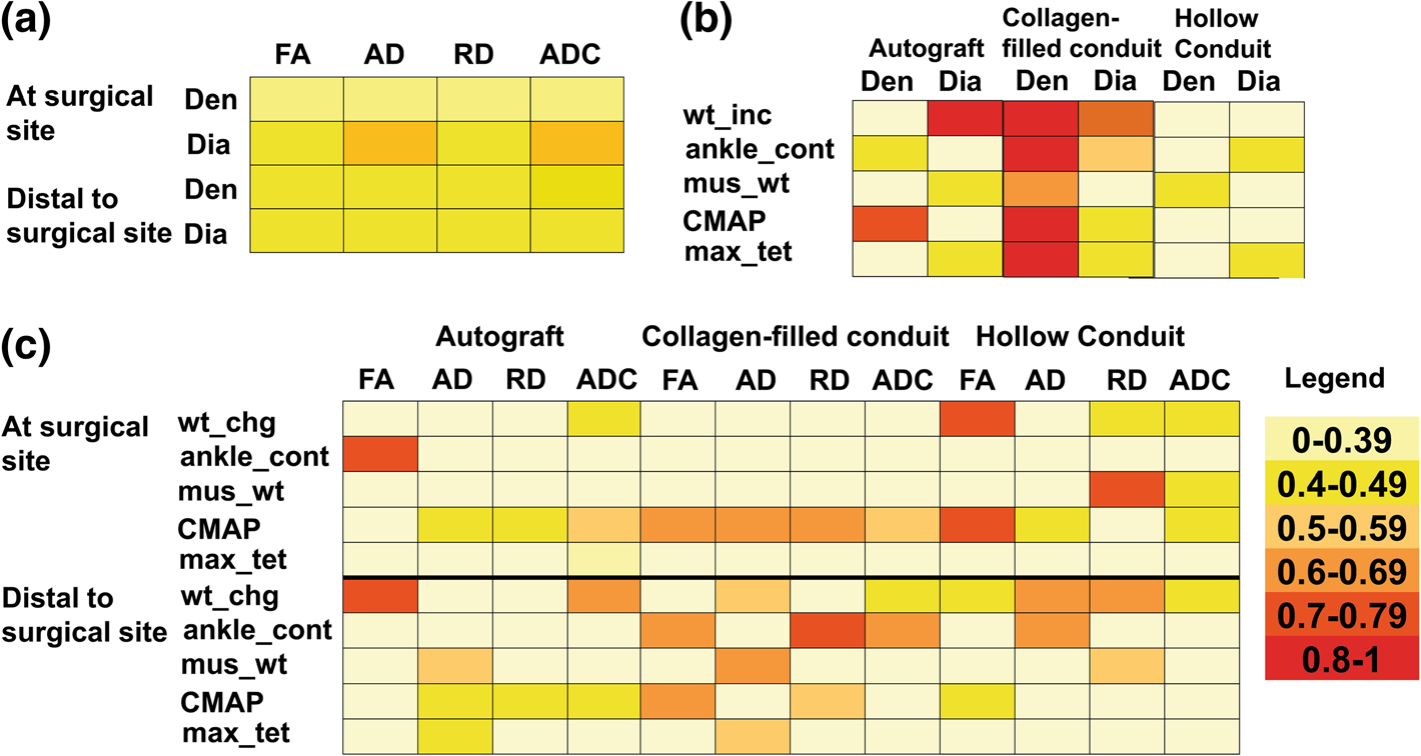
Correlation between DTI, functional measures, and histology. Spearman correlations of DTI and myelinated axon density and diameter (**a**), between histology and functional measures (**b**), and between DTI and functional measures (**c**) of the three groups. The legend (right panel) indicates the level of correlation, defined as weak (0.0–0.39), moderate (0.40–0.59), strong (0.60–0.79), or very strong (0.80–1.0). *AD* axial diffusivity, *ADC* apparent diffusion coefficient, *ankle_cont* ankle contracture, *CMAP* compound motor action potential, *Den* axonal density, *Dia* axonal diameter, *FA* fractional anisotropy, *max_tet* maximum tetanic force, *mus_wt* muscle weight, *RD* radial diffusivity, *wt_chg* weight change

There were moderate positive and negative correlations between axonal density and diameter and DTI metrics among all groups (Fig. 6a). The strongest positive correlations were found between AD and axonal diameter distal to the conduit site (*r* = 0.50), and for ADC and axonal diameter at the conduit site (*r* = 0.50). In addition, there were moderate correlations between functional measures and DTI metrics among all groups (Fig. 6b) and moderate to very strong negative correlations between functional measures and histology (Fig. 6c). Spearman’s correlation coefficients were not significant between DTI and histology and DTI and functional measures.

## Discussion

In our study, autograft facilitated the most sciatic nerve regeneration following segmental nerve surgery compared with collagen-based conduits. Significant group-level differences were only found with AD and axonal diameter distal to the surgical site, with autograft showing the greatest nerve regeneration, followed by hollow conduit and collagen-filled conduit, which may indicate that AD is potentially a viable DTI marker to detect group differences in axonal regeneration. Our results are corroborated by a prior ex-vivo rat study detecting a significant correlation of AD with axon diameter [13]. A possible explanation for this is the sensitivity of eigenvalues to underlying changes in axonal swelling and flow [14], which is abundant in axonal regeneration [15] and could have a direct impact on AD. In theory, water diffusion parallel to the axon is decreased due to a restriction in flow during injury.

Previous studies have shown in-vivo DTI to be predictive of nerve degeneration and regeneration in rabbit [16–18, 20] and rat [8–10] sciatic nerve crush and laceration injury models. These studies demonstrated an initial FA decrease and RD increase in the sciatic nerve distal to the injury site immediately following sciatic nerve crush injury, presumably due to Wallerian degeneration. Thereafter, there was a period of significant FA increase and RD decrease due to regenerating myelin sheath [8, 10, 16–18, 20] until reaching preinjury levels at 10 weeks. In our study, we did not find a significant RD decrease by week 13, which may indicate that the regenerative phase was incomplete at this time. Previous animal studies have shown that FA and RD correlate with the ratio of myelinated axons and total number of myelinated axons [8], which concur with DTI degenerative studies in the brain [21, 22].

Several animal model studies have shown that bioabsorbable nerve conduits are efficacious in facilitating regeneration across nerve gaps [23–28]. Prior studies examined the best combination of biomaterials to replicate autograft properties [29]. An ideal conduit should arguably include the presence of a basal lamina scaffold to serve as an adhesive and to promote dendrite elongation [30]. A semipermeable inner structure facilitates the entry of growth factors, secretion of neurotrophic factors by nerve stumps, and the exchange of metabolic nutrients [31]. The conduit should degrade at a rate long enough to warrant an environment in which regeneration and reorganization of the nerve is effective, but slow enough to deter scar tissue accumulation inhibiting longitudinal nerve growth [32, 33].

The Nerbridge™ collagen-filled conduit is a collagen-coated PGA conduit and porous collagen scaffold inner tube (collagen types I and III). It is biocompatible and commercially available for use in Japan. A previous rat study of a 1-mm transected laryngeal nerve bridged either with the collagen-filled conduit or direct suture assessed vocal fold mobility, nerve conduction velocity, morphology, and histology at 15 weeks following transection [34]. No significant differences in vocal fold recovery or conduction velocity were found in either group. However, more clearly myelinated fibers and less laryngeal muscle atrophy were observed for the collagen-filled conduit, supporting its potential efficacy. As evidenced in prior studies using conduits composed of PGA (the material of the collagen-filled outer core of the conduit), the structure tended to resorb quicker than other synthetic conduits [23, 35], further evidenced by a fully dissolved collagen-filled conduit at 13 weeks postsurgery. PGA-based conduits were superior to autograft in sensory recovery assessments in nerve gaps of less than 4 mm and greater than 8 mm [36]. A similar study in a rodent model involving much larger cohort sizes (without MRI) demonstrated overall similar results at the 12-week time point; however, analysis of a later 16-week time point revealed an accelerated rate of recovery in axonal density for collagen-filled conduit between 12 and 16 weeks postoperatively [37]. This suggests that the collagen-filled conduit may show improved recovery over the course of a longer time point, which was not investigated here.

Several reasons may help explain significantly larger axonal diameter and AD in the hollow conduit compared with the collagen-filled conduit in our study. One of the theoretical benefits of nerve conduits is to impede negative growth factors (e.g., excessive scar tissue, edema, inflammation, necrosis) from the internal, regenerating environment. Faster degradation rates of the collagen-filled conduit may be detrimental to nerve regeneration, as evidenced by the two hollow conduits still intact by week 13 compared with the collagen-filled conduit, all of which had dissolved. This could perhaps be specific to rabbits, due to their reliance on hopping and hind limb muscle function [38] which may result in proportionally larger amounts of scar tissue generated, although results of various transplants and regenerative successes in a rabbit and rat model were similar [39]. Rates of degradation as well as internal matrix morphology and pore size of conduits are areas that may warrant further exploration.

The extent of motor functional recovery was assessed by the change in maximum tetanic, CMAP, animal weight, ankle contracture, and muscle weight at the 13-week time point in the current study. Maximum tetanic is considered the most objective indicator of strength associated with the degree of muscle innervation by the nerve [40]. However, in this investigation, tetanic contraction had a high variance but did show favorability towards the collagen-filled conduit and conflicted with AD and axonal diameter findings. Tetanic-induced contractions provide a purely functional response, which could be originating from smaller axons if they form a tighter connection with muscle tissue. Hence, axon diameter may not accurately reflect actual function.

Several limitations to this study should be considered. First, due to the small diameter of the rabbit sciatic nerve (approximately 2–3 mm) and a relatively low in-plane spatial resolution of the echo-planar sequence (1.7 mm), partial volume effects at the borders of the nerve may have biased DTI measurements by averaging of voxels that include both nerve and surrounding scar tissue/muscle and may have falsely elevated ADC and lowered FA. Second, the study could have benefitted from the inclusion of more rabbits in each surgical group due to borderline *p* values after Bonferroni correction. Third, unlike previous studies, our investigation did not evaluate continuous time points, but rather a single time point that we thought would afford enough time for significant axonal regeneration based on previous crush and laceration studies demonstrating nearly complete sciatic nerve remyelination for a rabbit sciatic nerve by weeks 8–10 [16–18]. In our study, axon density and diameter did not return to expected levels as compared with the nonoperated nerve by the 13-week time point, indicating the healing process is likely longer for nerve gap versus laceration/crush injuries. It may be prudent in future studies to examine multiple time points following segmental nerve surgery and at a longer follow-up time to accurately determine the longitudinal time course of nerve regeneration with MRI, histology, and functional testing. Fourth, CMAP and maximum tetanic force are commonly used in research studies measuring functional recovery in rats [26, 27, 41], but we are unaware of such studies in rabbits. Additional functional measurements including the Tarlov scale, which assesses locomotor ability [42], and the toe-spreading reflex, which assess the degree of toe spread [43], have been evaluated in other rabbit studies [16–18, 20] and may have been useful to assess functional recovery.

In summary, autograft facilitated greater sciatic nerve regeneration following segmental nerve surgery compared with collagen-based conduits, hollow conduit, and collagen-filled conduit. Hollow conduit performed significantly better with regards to the axonal diameter and the DTI parameter of axial diffusivity compared with collagen-filled conduit. However, collagen-filled conduit performed better with regards to the maximum tetanic force, which is the most objective measure of functionality. This investigation suggests that DTI may be a potential, noninvasive biomarker to assess peripheral nerve regeneration. Ultimately, larger studies will need to be conducted to determine its efficacy and role.

## Additional file

Additional file 1: **Figure S1.** Whole nerve mean DTI measurements. There are no significant DTI differences between autograft (AG), collagen-filled (CF), and hollow conduit (HC), when measuring the proximal and distal sciatic nerve. (DOCX 2714 kb)

## Abbreviations

AD: Axial diffusivity
ADC: Apparent diffusion coefficient
CMAP: Compound motor action potential
DTI: Diffusion tensor imaging
FA: Fractional anisotropy
MRI: Magnetic resonance imaging
PGA: Polyglycolic acid
RD: Radial diffusivity
TA: Tibialis anterior
