# Supplementary material for: Evaluation of two collagen conduits and autograft in rabbit sciatic nerve regeneration with quantitative magnetic resonance DTI, electrophysiology, and histology
Source: Eur Radiol Exp. 2018 Aug 8;2:19. doi: 10.1186/s41747-018-0049-2 (PMC6091702; doi:10.1186/s41747-018-0049-2)
Supplement: Supplementary file 1 — Figure S1. Whole nerve mean DTI measurements. There are no significant DTI differences between autograft (AG), collagen-filled (CF), and hollow conduit (HC), when measuring the proximal and distal sciatic nerve. (DOCX 2714 kb) [file 41747_2018_49_MOESM1_ESM.docx]

**
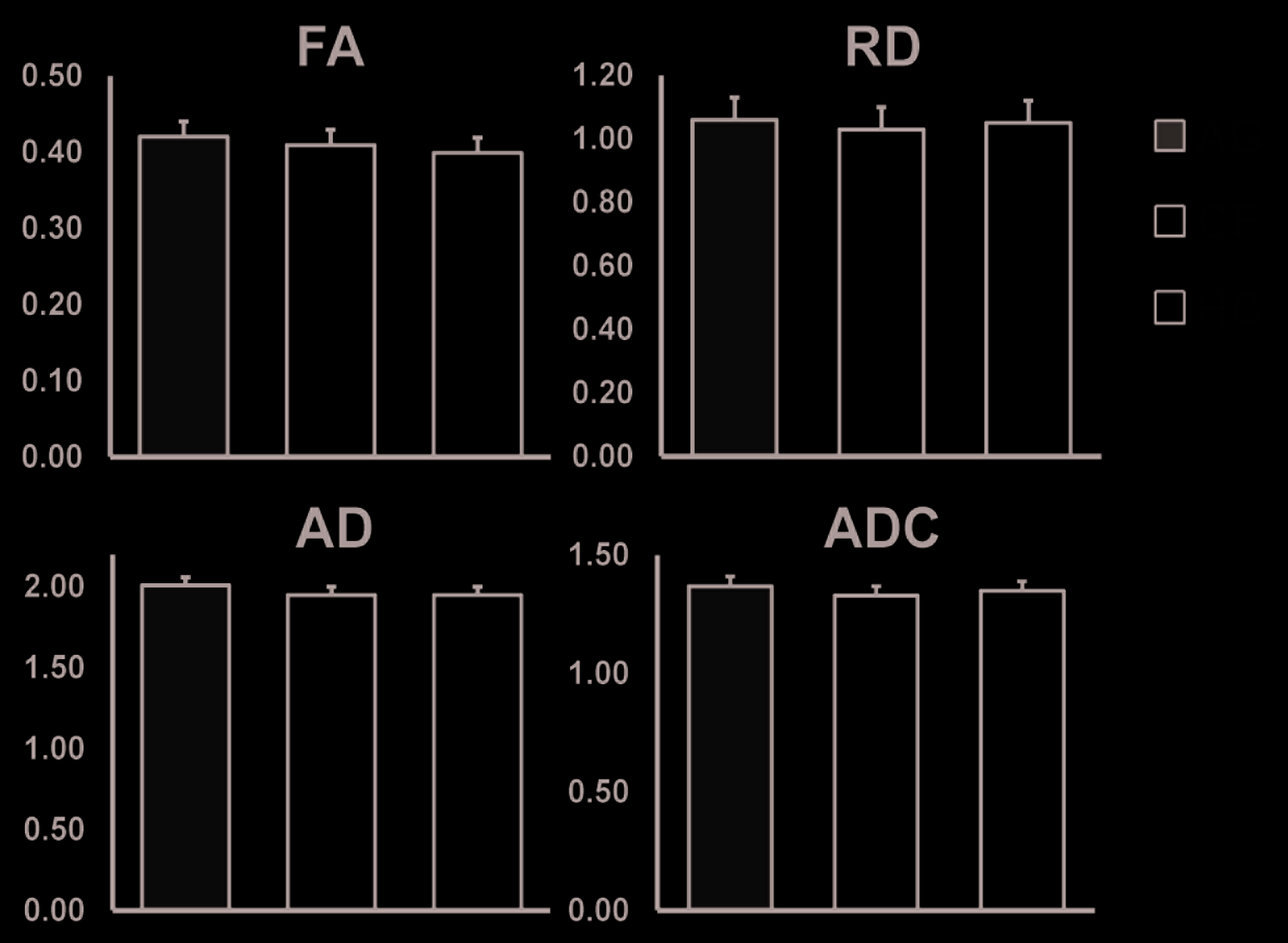
**

**Additional file 1: Figure S1.** Whole nerve mean DTI measurements. There are no significant DTI differences between autograft (AG), collagen-filled (CF), and hollow conduit (HC), when measuring the proximal and distal sciatic nerve.
